# The Chorioallantoic Membrane as a Platform for Developing Vascularized Cell Macroencapsulation Devices

**DOI:** 10.1155/term/5577199

**Published:** 2025-11-15

**Authors:** Murillo D. L. Bernardi, Sonny F. de Jong, Maarten B. Rookmaker, Andrej Shoykhet, Roel Deckers, Silvia M. Mihăilă, Rosalinde Masereeuw, Marianne C. Verhaar

**Affiliations:** ^1^Department of Nephrology, University Medical Center Utrecht, Utrecht, the Netherlands; ^2^Division of Imaging & Oncology, University Medical Center Utrecht, Utrecht, the Netherlands; ^3^Division of Pharmacology, Utrecht Institute for Pharmaceutical Sciences, Utrecht University, Utrecht, the Netherlands

**Keywords:** angiogenesis, bioartificial kidney, macroencapsulation, tissue engineering, vascularization

## Abstract

**Background:**

Cell macroencapsulation devices (CMD) offer a promising solution for organ function replacement by shielding implanted cells from the host immune system while allowing the exchange of nutrients and waste products. Developing efficient CMD necessitates optimizing vascular integration, membrane permeability, and cellular functionality using robust preclinical models. In this study, we adapted the chick chorioallantoic membrane (CAM) model to develop and evaluate CMD.

**Methods:**

Semipermeable membranes were integrated into the CAM, with vascularization modulated through growth factors and extracellular matrix manipulation. Human kidney tubular epithelial cells were cultured on these vascularized membranes to assess cell viability, polarization, and functionality, including selective transport and barrier integrity.

**Results:**

The membranes integrated successfully into the CAM and supported functional vascularization, demonstrating selective permeability by facilitating the exchange of low-molecular-weight compounds while preventing the infiltration of larger proteins and cells, thereby creating an immune-isolated environment. Kidney tubular epithelial cells remained viable, polarized, and functionally active, showcasing selective compound transport and robust barrier integrity.

**Conclusion:**

These findings underscore the CAM model's utility in evaluating vascular integration, membrane permeability, and epithelial cell functionality, all critical parameters for CMD development. The CAM model provides a rapid, cost-effective platform for CMD assessment, significantly accelerating their development and potential clinical translation. This approach holds particular promise for applications targeting kidney diseases characterized by compromised transport functions, offering a pathway toward more effective therapeutic solutions.

## 1. Introduction

The increasing prevalence of organ failure presents a growing global health concern, driving the search for more effective and accessible treatment options. While existing solutions, such as mechanical organ support and transplantation, can prolong life, they often fall short of restoring the intricate biological functions of the affected organs [1]. The limitations of these approaches, including donor shortages and risks of immune complications, highlight the need for innovative strategies to address organ failure [2–4].

Cell and tissue therapies have received increasing attention in recent decades. Developments in stem cell biology have shown interesting options, particularly through the use of induced pluripotent stem cells (iPSCs) [5–7]. Although tissues and organs can be grown from iPSCs, these tissues are usually immature and their use is complicated by off-target differentiation and even teratogenic degeneration [8]. Hybrid solutions combining cells with physical components are also under development such as the bioartificial kidney [9]. Here, extracorporeal organ replacement therapy is combined with epithelial cells that add metabolic functions and additional clearance to the passive diffusion of dialysis. A major drawback of this approach is the need for access to the large vasculature with all its vascular and infection risks.

Cell macroencapsulation devices (CMD) are a promising alternative in the treatment of organ failure. These devices enclose therapeutic cells with a semipermeable membrane that not only guide the architecture and function of bioengineered cells but also serve as an immune barrier, shielding the cells from host attack and preventing undesirable cell migration or infiltration [10–15]. These permeable membranes still allow the exchange of nutrients, metabolic waste, and signaling molecules between the encapsulated cells and the host environment—features critical for sustaining cellular function and achieving therapeutic outcomes [16]. CMD are particularly promising for treating conditions like kidney failure, where the loss of transport, filtration, and hormonal functions severely impacts patient outcomes. By integrating functional epithelial cells within these devices, CMD have the potential to restore selective transport and metabolic functions that current treatments, such as dialysis, cannot replicate.

In the development of CMD, optimization of the membrane plays a crucial role as it forms the interface between bioengineered tissue and the recipient. The successful development and integration of the membrane involve several steps: implantation, establishment of a functional connection to the host vasculature, efficient exchange of substances over the membrane and survival of the bioengineered tissue, while retaining functionality. The use of hydrogels and growth factors can enhance vascularization, as they provide a supportive extracellular matrix–like environment that promotes angiogenesis and vessel formation around the implanted constructs [17, 18]. In vivo validation is essential for assessing these parameters, particularly in early-stage development, yet conventional rodent models are costly, time-consuming, and raise ethical constraints [19]. A high-throughput, ethically favorable alternative could significantly accelerate CMD development.

The chick chorioallantoic membrane (CAM) model is an established in vivo system known for its robust vascularization and ease of manipulation [20]. Historically, the CAM model has been a valuable tool to investigate and modulate vascularization as it has been shown in various research applications, including cancer studies [21, 22], angiogenesis research [23], and drug testing [24]. The CAM model offers several advantages over traditional animal models. It is comparatively of low cost and straightforward to use, requiring minimal specialized equipment and facilities. CAM experiments can be conducted under standard cell culture conditions, yet they enable the study of biological behavior, such as angiogenesis, tissue remodeling, and vascular interactions, that cannot be replicated in vitro.

The CAM model offers valuable insights into how vascularized environments influence the behavior and function of encapsulated cells, especially epithelial cells relevant to organ-like functions. However, its use for studying epithelialized semipermeable membranes, such as those used in CMD, remains underexplored. In particular, the ability of epithelial monolayers to maintain polarity, barrier integrity, and selective function within a vascularized context is critical for CMD functionality, but poorly characterized. This is a critical aspect in the development of CMD, especially for applications requiring directional transport across epithelial barriers (e.g., kidney tubules, intestine, or liver).

Here, we present a strategy to evaluate CMD performance using the CAM model. We integrate cell culture inserts as CMD-like platforms into the CAM system, enabling the study of epithelial cell behavior, membrane barrier integrity, and molecular transport in a vascularized context. Additionally, we provide tools for optimizing vascularization through comprehensive histological and functional analyses, including the diffusion assessment of circulating compounds of various molecular sizes. As a proof of concept, we seeded conditionally immortalized proximal tubule epithelial cells (ciPTEC) in the device to evaluate their postimplantation survival, epithelial integrity, and functionality, focusing on compound-specific transport activity.

## 2. Methods

### 2.1. *Ex Ovo* CAM Assay

Fertilized Leghorn chicken eggs (Drost Loosdrecht B.V., *Loosdrecht, the Netherlands*) were used in all in vivo experiments. Preincubation and incubation were performed according to the protocol as previously described [25]. Briefly, on embryonic Day 0 (ED0), eggs were placed in a humidified incubator at 37°C with 75% relative humidity and rotated 12 times per day. On ED3, eggshells were disinfected with 20% ethanol, and the embryos were transferred to sterilized polystyrene weighing boats (VWR International*, Radnor, PA, USA*), each placed within a standard 100-mm Petri dish (Greiner Bio-One GmbH, Kremsmünster, Austria). The weighing boats were then incubated at 37°C with 0% CO_2_ and relative humidity > 90% for the remainder of the experiment. Embryo viability was monitored daily; nonviable embryos were removed promptly to prevent contamination. On ED10, BRAND 2-in-1 cell culture inserts for 24-well plates, featuring a polyester membrane with 0.4-μm pores and a surface area of 0.66 cm^2^ (BRANDTECH Scientific, Wertheim, Germany), were placed onto the CAM for vascularization. Per embryo, one insert was used unless mentioned otherwise. Experimental procedures were performed on ED14, after which the embryos were euthanized by decapitation, a method recognized as humane and recommended for minimizing distress in avian models [26]. In total, 54 embryos were used across all experiments in this study. Specific numbers used for each experiment are detailed in the respective subsections.

### 2.2. Assessment of the CAM Vasculature

#### 2.2.1. Scanning Electron Microscopy (SEM)

SEM was performed on the CAM from two embryos. The CAM were collected and fixed with 4% paraformaldehyde. The tissue was embedded in paraffin blocks, which were then sectioned into 3-μm-thick slices and mounted on 10-mm glass cover slips. The mounted samples were deparaffinized in Histo-Clear (National Diagnostics, Atlanta, GA, USA) twice for 5 min each. Following deparaffinization, the samples were rehydrated through a graded ethanol series: 100%, 90%, and 70%, and distilled water, each for 3 min twice.

After rehydration, the samples were rinsed with PHEM buffer (Sigma-Aldrich, Merck KGaA, Darmstadt, Germany) twice for 2 min. Post-rinsing, the samples were dehydrated through a graded ethanol series: 70%, 80%, 90%, and 100%, each for 10 min. Subsequently, the samples were exposed to 100% hexamethyldisilazane (HMDS) (Sigma-Aldrich, Merck KGaA, Darmstadt, Germany) for 3 min. Excess HMDS was blotted off, and the samples were dried in a desiccator for 25 min.

The dried samples were mounted on 10-mm stubs using carbon tape to enhance adhesion and conductivity. They were then coated with a 6-nm layer of gold using a Rotary Pumped Coater (Q150R S, *Quorum Tech, Lewes, UK*). Imaging was performed using the FEI SCIOS system (Thermo Fisher Scientific, *Eindhoven*, the Netherlands) with the T2 detector and OptiTilt use case to capture detailed morphological features of the CAM vasculature.

#### 2.2.2. Hematoxylin and Eosin (H&E) Analysis

To assess the vasculature of the CAM, a piece of the CAM was excised from one embryo and processed for histological analysis, which included embedding the tissue in optimal cutting temperature (OCT) compound, followed by cryosectioning into 10-μm-thick sections using a cryostat. These sections were subsequently mounted on glass slides for H&E staining.

Initially, the slides were fixed in 4% paraformaldehyde for 15 min at room temperature (RT). After fixation, the slides were rinsed in distilled water and then stained with hematoxylin for 5 min. Excess hematoxylin was removed by rinsing the slides under running tap water for 5 min. The sections were then differentiated in 1% acid alcohol for 30 s and blued in Scott's tap water substitute for 1 min. Following this, the slides were stained with eosin for 2 min, then dehydrated through graded alcohols (70%, 95%, and 100% ethanol), cleared in xylene, and mounted with a coverslip using a resinous medium. The stained sections were examined under a light microscope to evaluate the vascular structures within the CAM.

#### 2.2.3. Super-Resolution Ultrasound (SRUS) Imaging

For SRUS imaging, 3 fertilized chicken eggs were opened on ED3, and their contents were transferred to sterilized, modified weighing boats (VWR International, Amsterdam, the Netherlands). One side of each weighing boat was replaced by a vertical Mylar film (thickness 46 μm; Goodfellow Cambridge Ltd., Huntingdon, UK), which was affixed using a silicone adhesive (Dowsil 732, Dow Inc., Midland, MI, USA). The Mylar wall enabled lateral ultrasound imaging and permitted vertical movement of the transducer without disturbing the embryo. The embryos in these modified boats were incubated in a humidified incubator at 37.5°C until ED12.

For this study, SRUS imaging was performed on three embryos. Prior to imaging, the CAM was injected with 50 μL of SonoVue microbubble (MB) contrast agent (Bracco Imaging S.p.A., Milan, Italy), prepared according to the manufacturer's instructions. This resulted in an initial MB concentration of 2 × 10^8^ MB/mL.

US acquisitions were performed using a Verasonics Vantage System (Verasonics Inc., Kirkland, WA, USA) with an L11-5v transducer at a center frequency of 7.6 MHz for transmit pulses. High-frame-rate plane-wave B-Mode imaging was performed with 5-angle (−6°, −3°, 0°, 3°, 6°) coherent compounding and a postcompounding frame rate of 500 Hz. The transducer was mounted on a motorized platform, allowing precise three-dimensional translation.

Following image collection, the SRUS images were realized using a postprocessing pipeline consisting of several steps: image filtering, MB detection, localization, and tracking. The SRUS postprocessing scripts were adapted from Heiles et al. [27] and Denis et al. [28], with an additional step for noise equalization introduced by Song et al. [29]. First, B-mode image reconstruction from radio-frequency to intensity data, including delay and sum beamforming and spatial compounding, was performed using Verasonics software. Next, filtering was employed to suppress tissue signals and optionally filter MB speed, with noise equalization applied via a 1D noise profile. Localization was then carried out by detecting local maxima, with a radial symmetry algorithm applied to a 5 × 5 pixel window around the brightest maxima. Finally, localized MBs were tracked across frames using the simpletracker algorithm [30], and the detected tracks were rendered into a high-resolution image with a pixel size five times smaller than the wavelength of the transmitted. Furthermore, super-resolved flow velocity and direction maps were generated.

### 2.3. Vasculature Manipulation

We tested different hydrogels to increase vascular density and leakiness in the CAM, aiming to enhance its suitability as a model for studying vascularized devices and angiogenesis-related processes. Twenty-one embryos were used in these experiments.

#### 2.3.1. Hydrogel Preparation

Fibrin hydrogels were prepared by dissolving fibrinogen (10 mg/mL Sigma-Aldrich, Merck KGaA, Darmstadt, Germany) in phosphate-buffered saline (PBS). Rat-tail collagen Type I (2 mg/mL; Sigma-Aldrich) preadjusted to pH 7.1, was pipetted into 50-μL droplets, and allowed to polymerize in a Petri dish at 37°C for 30 min. Collagen-fibrinogen (COL-FIB) composite hydrogels were prepared by mixing fibrinogen (7.5 mg/mL) with collagen I (2 mg/mL) in PBS, followed by incubation at 37°C for 30 min. Cultrex Basement Membrane Extract (BME) (R&D Systems, Bio-Techne Ltd, Abingdon, UK) was pipetted in 50-μL droplets and similarly gelled at 37°C for 30 min. When required, 0.5 μg of recombinant human vascular endothelial growth factor (VEGF; 500 ng/gel; PeproTech, London, UK) was added to the hydrogel mixture prior to gelation.

#### 2.3.2. Hydrogel Placement and Analysis

The gels were placed on top of the CAM of the embryos on ED7. Three hydrogels were placed per embryo. After 7 days, images of the gels were taken using a stereomicroscope. The captured images were pre-processed using FIJI software [31] (ImageJ, National Institutes of Health, Bethesda, MD, USA) to enhance contrast and clarity. Angiogenic-related parameters, including total vessel length (TVL) and total number of junctions (NVJ), were quantified using AngioTool software [32] (version 0.6a, National Cancer Institute, Bethesda, MD, USA). The vasculogenic index, defined as the number of visible vessels radiating from the hydrogel's center with an angle of ≤ 45° [33–35], resembling a spoke-wheel pattern, was determined through manual counting by two independent, blinded observers to ensure reproducibility.

### 2.4. Connecting (Cellularized) Membranous Devices to the CAM Vasculature

The devices used were BRAND 2-in-1 cell culture inserts for 24-well plates, featuring a polyethylene terephthalate (PET) membrane with 0.4-μm pores, pore density of 2 × 10^6^/cm^2^, and a surface area of 0.66 cm^2^ (cat 782711, BRANDTECH Scientific, Wertheim, Germany). These inserts were chosen due to their practical handling characteristics and the presence of a semipermeable membrane suitable for cell seeding. To enhance stability and prevent tilting on the CAM, a 3D-printed circular support (15-mm diameter) made of polylactic acid (PLA) was used to secure the inserts during implantation.

#### 2.4.1. Pore Selectivity Analysis

To evaluate the integrity and pore selectivity of the device membrane (0.4 μm), cellular diffusion analyses were performed after a 4-day culture period in three embryos. Inserts with intact membranes were compared to inserts with compromised membranes, which were pierced five times using a 30G needle. After culture, the contents of the inserts were collected, centrifuged to pellet any cells, and the resulting pellet was assessed using a Neubauer chamber for quantification. Multiple fields were analyzed for each condition to ensure accurate comparison.

#### 2.4.2. Diffusion Experiments

A total of 12 embryos were used in the diffusion experiments. The inserts were placed on the surface of the CAM at ED10, when the CAM fully covered the Petri dish. Inserts remained in place for 4 days. For the VEGF experiments, two inserts with 200 μL of PBS were placed on opposite sides of the same embryo with the supporting ring, with 0.5 μg/mL VEGF added to one insert. On ED14, embryos were injected into superficial CAM vessels with 200 μL of a 2-mg/mL solution of 10-kDa Dextran-FITC (Sigma Aldrich, *Amsterdam, the Netherlands*) and returned to the incubator for the remainder of the diffusion experiment (30 min–2 h). Samples were collected from the supernatant at 30-min intervals, and relative fluorescence intensity was measured using a CLARIOstar Plus fluorimeter (BMG Labtech, *Ortenberg, Germany*).

#### 2.4.3. ciPTEC Experiments

Human ciPTEC transduced with the organic anion transporter 1 (OAT1) were obtained from Cell4Pharma (Oss, the Netherlands) and cultured in phenol-red-free DMEM-HAM's F12 medium (Lonza, Basel, Switzerland) supplemented with 10% (v/v) fetal calf serum (FCS, Greiner Bio-One GmbH, Frickenhausen, Germany), insulin–transferrin–sodium selenite media supplement (Sigma-Aldrich, Merck KGaA, Darmstadt, Germany; cat. no. 11074547001, insulin 5 μg/mL; transferrin 5 μg/mL; sodium selenite 5 ng/mL), 36-ng/mL hydrocortisone (Sigma-Aldrich, Merck KGaA, Darmstadt, Germany; cat. no. H0135), 10-ng/mL epidermal growth factor (Sigma-Aldrich; cat. no. E9644), 40-pg/mL 3-iodothyronine (Sigma-Aldrich; cat. no. T5516), 10% (v/v) fetal bovine serum (FBS, Gibco, Thermo Fisher Scientific, Paisley, UK, cat. no. 16140-071, or Greiner Bio-One, Alphen aan den Rijn, the Netherlands, cat. no. 758093), and 1% (v/v) penicillin/streptomycin (Sigma-Aldrich; cat. no. P4333), as reported [36, 37]. Cells were expanded at 33°C until confluency and then harvested using Accutase (Sigma-Aldrich, Merck KGaA, Darmstadt, Germany). Membranes were double-coated with L-DOPA (2 mg/mL, Sigma-Aldrich) and collagen IV (25 μg/mL, Corning Inc., Corning, NY, USA) prior to seeding with 50,000 cells in 200 μL of expansion medium per insert. Inserts were maintained at 33°C for 5 days for expansion and differentiated at 37°C for 7 days, with the last 4 days on the CAM. Inserts were placed in vascularized areas of the CAM with the 3D-printed PLA stabilizers and kept with 200 μL of their culture media, refreshed every 2 days. A total of 12 embryos were used for these experiments.

#### 2.4.4. Cell Barrier Integrity

Two inserts per CAM were used: one seeded with ciPTEC and one empty (control). Inserts were filled with 200 μL of Hanks' Balanced Salt Solution (HBSS, Lonza, Basel, Switzerland), and 1-mg/mL inulin-FITC (Sigma-Aldrich, Merck KGaA, Darmstadt, Germany) was injected onto the CAM using a 30G needle. After 60 min, supernatants were collected, and fluorescence intensity was measured with a CLARIOstar Plus fluorimeter (BMG Labtech, Ortenberg, Germany; excitation 495 nm, emission 515 nm).

#### 2.4.5. Vascular Staining and Cellular Survival

ciPTEC viability and the underlying vasculature were assessed after 4 days of culture on the CAM using calcein-AM staining. Briefly, cells were incubated with 500 μL of 2 μM calcein-AM (Thermo Fisher Scientific, Roskilde, Denmark) in serum-free medium, added to the apical side of the insert, for 30 min at 37°C. Simultaneously, embryos were injected with 200 μL of LCA-Rhodamine (Vector Laboratories, Susteren, the Netherlands; 1:20 in PBS) to stain the vasculature. After a 10-min circulation period, the CAM was fixed in 4% formaldehyde (Sigma-Aldrich, Merck KGaA, Darmstadt, Germany) for 20 min. Following fixation, inserts were gently washed three times with PBS to remove excess dye. The membrane of the insert, along with the attached CAM, was excised using an 8-mm biopsy punch and transferred to a fresh plate containing PBS. Samples were immediately visualized using confocal microscopy (Leica Microsystems, Wetzlar, Germany), capturing images in both red and green channels for detailed visualization and analysis. Immunostainings were performed on ciPTEC to assess cell polarity, targeting cilia with acetylated tubulin, the cytoskeleton with phalloidin, and DNA with DAPI. Cells were fixed with 4% formaldehyde (Merck, Darmstadt, Germany) for 45 min at RT. This was followed by permeabilization and blocking using a solution of 0.5% Triton X-100 (Merck, Darmstadt, Germany) and 0.5% bovine serum albumin (BSA, Sigma-Aldrich) in PBS for 30 min at RT. After a single wash with 0.5% BSA in PBS, primary antibodies were added in 0.5% BSA in PBS: mouse anti-acetylated α-tubulin (1:200, Sigma-Aldrich) and incubated overnight at 4°C. Cells were then rinsed once with 0.5% BSA + 0.1% Tween 20 in PBS, followed by the addition of secondary antibodies and counterstains in 0.5% BSA + 0.1% Tween 20 in PBS: Rhodamine-Phalloidin (Thermo Fisher Scientific), Alexa Fluor 647 donkey anti-mouse IgG (1:300, Invitrogen, Thermo Fisher Scientific, Paisley, UK), and DAPI (1:1000, Merck, Darmstadt, Germany). The cells were incubated with these reagents for 2 h at RT, then washed twice with 0.5% BSA + 0.1% Tween 20 in PBS. Finally, the samples were mounted using VectaShield mounting medium (Vector Laboratories, Susteren, the Netherlands) on glass slides and imaged using a confocal microscope (Leica Microsystems, Amsterdam, the Netherlands).

#### 2.4.6. Functional Transport

ciPTEC-seeded inserts were kept in the CAM for 4 days, with two inserts used per embryo. One insert was pretreated with efflux pump inhibitors, specifically 20 μM MK571 (Tocris Bioscience, Bristol, UK) and 20 μM KO-143 (Tocris Bioscience, Bristol, UK), in HBSS, for 30 min, while the other insert was exposed to HBSS alone, serving as control. Embryos were injected with 0.2 mL of 200 μM fluorescein (Sigma-Aldrich, Merck KGaA, Darmstadt, Germany), a substrate for OATs [37]. Inserts were removed after 1 h, washed with PBS, lysed with 1 M NaOH, and fluorescence intensity was measured using a CLARIOstar Plus fluorimeter (BMG Labtech, Ortenberg, Germany; excitation 495 nm, emission 515 nm).

### 2.5. Data Analysis & Statistics

All experimental data were analyzed using GraphPad Prism software (GraphPad Software*, San Diego, CA, USA*). Statistical comparisons were performed using one-way or two-way analysis of variance (ANOVA), as appropriate, followed by Tukey's post hoc test for multiple comparisons. Data are presented as mean ± standard deviation (SD) unless otherwise indicated. Significance levels were set at *p* < 0.05. All analyses were performed on at least three biological replicates, with technical replicates included to ensure data robustness. Sample sizes and specific statistical tests applied to individual experiments are detailed in the respective figure legends.

## 3. Results

### 3.1. CAM Model Enables Qualitative and Quantitative Evaluation of Vascularization

**Figure 1:**
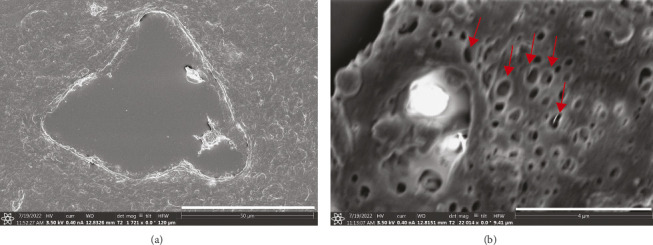
Representative SEM images of the CAM. (a) Cross-sectional view of a CAM capillary, showing its structural organization. (b) Higher-magnification image of the endothelial lining, highlighting the presence of fenestrae-like structures with red arrows. Embryo at ED14. Scale bar (a): 50 μm, (b): 4 µm.

Qualitative assessment of the vascularization in the CAM was performed on the *ex ovo* CAM assay. After transferring the embryos to Petri dishes, heart activity was visually detected in up to 70% of the embryos, indicating a high rate of viability post-transfer. The embryos continued to develop well in the dish, surviving in the incubator up to at least Day 14, when experiments were terminated. Gross examination revealed progressive vascular expansion between ED5 and ED10, with full coverage of the Petri dish surface by ED10 (Supporting Figure 1). Histological analysis by H&E staining confirmed the presence of a dense-vascular network and organized endothelial cell layer within an organized stroma. SEM provided detailed morphological insights, showcasing capillaries with continuous endothelial lining and the presence of erythrocytes within their lumens (Figure 1(a)), as well as fenestrae-like structures on the endothelium (Figure 1(b)), indicative of a functional and semipermeable vasculature.

**Figure 2:**
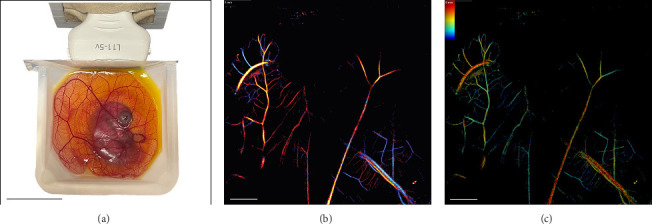
Capillary patency evaluation of the CAM via SRUS imaging following microbubble injection. (a) Setup illustrating the ex ovo cultured chicken embryo in its container and the ultrasound probe used for imaging. (b) Representative images of super-resolved flow direction map of the CAM microvasculature, where red indicates flow toward the transducer and blue indicates flow away from the transducer. (c). Representative flow velocity map, with warmer colors (red) representing faster flow and cooler colors (blue) representing slower flow. Scale bars: (a) 7 cm, (b) and (c) 5 mm.

In addition to qualitative assessment, we established functional and quantitative evaluations of vascularization in the CAM. To ensure the presence of a closed circulation with distinct arterial and venous components, we utilized SRUS imaging [38, 39]. Directional flow and velocity maps revealed perfused vessels with distinguishable arterial and venous components (Figures 2(a), 2(b), and 2(c)), confirming a closed and dynamic vascular network. High-resolution tracking of MB movement validated continuous blood flow throughout the CAM. Functional microscopy further confirmed these findings, visualizing individual erythrocytes flowing within the CAM (Supporting Video 1).

**Figure 3:**
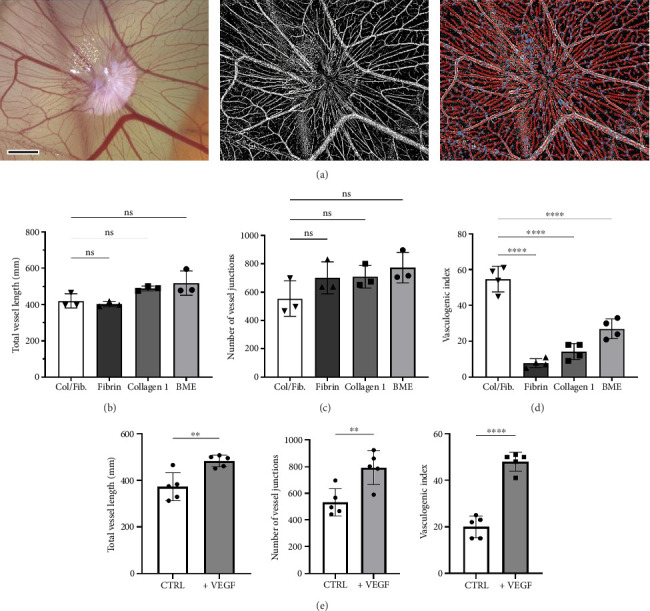
Quantitative analysis of CAM vascularization in response to hydrogel composition and growth factor supplementation. (a) Schematic illustrating the workflow for automated quantification of vascular parameters using the AngioTool software. Captured images of the hydrogel samples are processed to generate binary representations, which are then analyzed to determine the total vessel length (TVL) and total number of vessel junctions (TNJ). Scale bar = 2 mm. (b–d) Graphs showing the (b) TVL, (c) TNJ, and (d) vasculogenic index (vessels/mm) for the various hydrogel conditions tested, including collagen I (COL-1), fibrin (FIB), and the combination of collagen I and fibrinogen (COL-FIB). (e) Comparison of TVL, TNJ, and vasculogenic index between the COL-FIB hydrogel with and without the addition of vascular endothelial growth factor (VEGF). Statistical analysis was performed using ANOVA, with results presented as mean ± SD. Significance levels are denoted as ns = *p* > 0.05; ^∗^*p* < 0.05; ^∗∗^*p* < 0.01; ^∗∗∗^*p* < 0.001; ^∗∗∗∗^*p* < 0.0001. Sample sizes (*n*) range from 3 to 5.

The vascular density of the CAM can be structurally evaluated using computational tools, assessing angiogenesis parameters such as TVL, NVJ, and the vasculogenic index (Figure 3(a)). The images revealed a highly organized and densely vascularized network, comprising vessels of varying calibers and branching orders. Larger primary vessels gave rise to smaller secondary and tertiary branches, culminating in a rich capillary plexus. This hierarchical structure, with observable sprouting, reflects active angiogenic remodeling consistent with sprouting angiogenesis, a well-documented phenomenon in CAM literature. Such complexity underscores the CAM's suitability for assessing vascular dynamics and network maturation in engineered constructs.

As vascularization is crucial for both CMD cell survival and functionality, the effects of various extracellular matrices hydrogels (collagen Type I, fibrin, BME, and COL-FIB) and growth factors (VEGF) on CAM vascularization were evaluated. While TVL and NVJ were not significantly different among the all hydrogels formulations (Figures 3(b) and 3(c)), the COL-FIB hydrogel induced a significantly enhanced vasculogenic index (22.6 ± 4.8 vessels/mm^2^) compared to fibrin (7.2 ± 2.5) and collagen Type I (9.6 ± 1.5) (*p* < 0.0001, Figure 3(d)). Based on these findings, we continued our experiment with the COL-FIB hydrogel formulation and supplemented with VEGF to further promote angiogenesis.

The addition of VEGF increased all vascular parameters: the TVL rose from an average of 372.5 ± 60.2 mm to 483.3 ± 25.1 mm (*p* < 0.01), NVJ increased from 534 ± 102 to 793 ± 125 junctions (*p* < 0.01), and the vasculogenic index doubled to 48.0 ± 4.0 vessels/mm (*p* < 0.05) compared to the non-VEGF condition (Figure 3(e)).

### 3.2. Membranes Integrate With the CAM Vasculature and Allow Compound Diffusion

**Figure 4:**
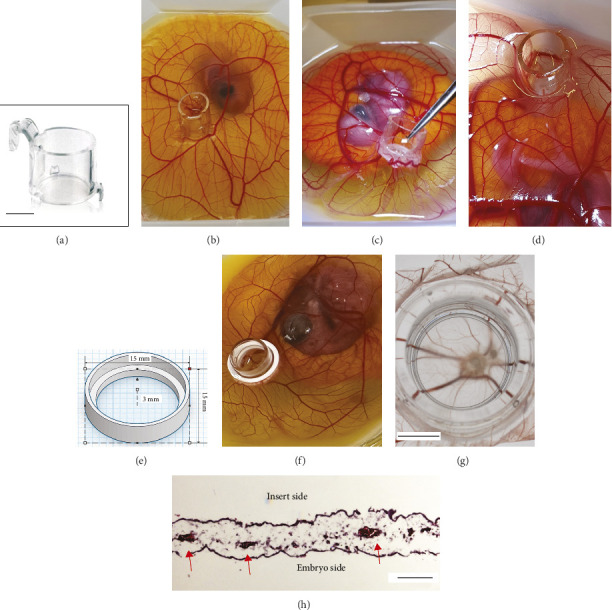
Experimental setup for integrating membrane devices into the CAM. (a) BRAND 2-in-1 24-well cell culture insert with supporting feet, featuring an 8.8-mm semipermeable membrane (0.4 μm pore size), used as the membrane device. Scale bar: 4 mm. (b) Placement of the device on the CAM on embryonic Day 10, positioned over major blood vessels. (c) Device after 4 days, showing vascularization and integration with the CAM tissue. (d) Some devices sunk into the CAM, leading to leakage of egg materials over the top of the insert. (e) 3D schematic of the supporting structure to hold the insert in place. (f) Placement of the insert with the supporting device within the CAM. (g) Stereomicroscopy of the insert and the underlying CAM, revealing multiple large and small vessels directly beneath the semipermeable membrane. Scale bar: 5 mm. (h) Histological cross-section of the CAM, arrows point to the capillaries less than 50 microns from the semipermeable membrane in the cell culture insert. Scale bar: 100 µm.

To secure the membranous device to the CAM vasculature and prevent tilting, sinking, or sliding, proper fixation was essential. Initially, tilting led to egg contents entering the inserts from above (Figures 4(a), 4(b), 4(c), and 4(d)). To resolve this, we developed a custom 3D-printed stabilizer (Figure 4(e)) that provided the necessary mechanical support. With this stabilizer, we successfully positioned the insert on the CAM at ED10, ensuring stability throughout the following 4-day incubation period (Figures 4(f) and 4(g)). After this period, the CAM attached strongly to the insert, resisting even the insert being pulled with a tweezer (Figure 4(c)). Histological analysis of the excised CAM and membrane through cross-sectional H&E staining revealed the presence of multiple capillaries distanced within 50 μm from the semipermeable membrane (not shown in the staining), demonstrating successful vascular approximation (Figure 4(h)). Additionally, embryo survival remained high, with a rate of approximately 70% maintained until the end of the experiment.

To confirm that the 0.4-μm semipermeable membrane functioned as an effective barrier by preventing cells from the embryo from passing through during the experimental period, we assessed how the membrane integrity affects diffusion of blood cells from the CAM to the inserts. This involved comparing inserts with intact membranes to those deliberately compromised by piercing the membrane five times with a 30G needle. In devices with intact membranes, no cells were detected within the insert after the 4-day culture period. In contrast, inserts featuring intentionally punctured membranes averaged 112 ± 35 cells per image field (Supporting Figure 2).

**Figure 5:**
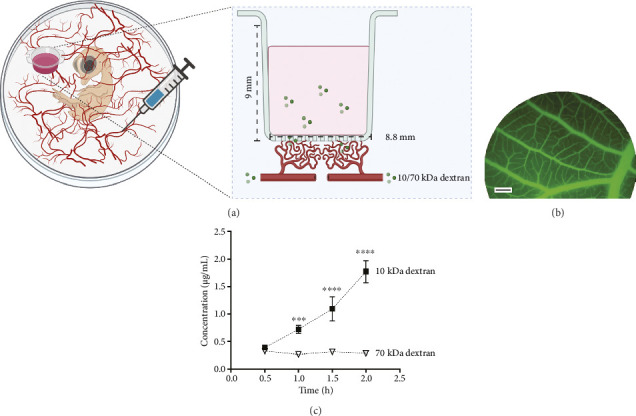
Dextran diffusion assay in the CAM model. (a) Experimental setup for the dextran diffusion assay. A 0.2-mL solution of 1-mg/mL, 10-kDa FITC-labeled dextran is injected into a large vein distant from the cell-free device and embryo's body. The fluorescent dextran molecules extravasate the vasculature adjacent to the device and diffuse through the porous membrane, accumulating in the PBS within the insert. (b) Overview of the CAM showing large and small vessels in green after dextran injection. Scale bar = 500 μm. (c) The concentration of the 10-kDa dextran increases at a much higher rate over time compared to the 70-kDa dextran on the inserts, as shown by the black and white symbols, respectively. (One-way ANOVA, mean ± SD. ^∗∗∗^*p* < 0.001; ^∗∗∗∗^*p* < 0.0001. *n* = 3 samples per condition and time point).

The functionality of the vascular connection to the membranous device was evaluated by injecting fluorescently labeled dextran of different sizes into the embryo's circulation and measuring their accumulation in the inserts (Figure 5(a)). Dextran was immediately and evenly distributed in the vessels of the CAM, confirming effective delivery throughout the vascular network (Figure 5(b)). Diffusive transfer of dextran from the blood vessels into the inserts increased over time, indicating effective compound exchange between the vasculature and the device. Size selectivity in molecular transport was explored by comparing the diffusion rates of 10-kDa and 70-kDa dextran-FITC. Within 1 hour, the diffusion rate of 10-kDa dextran was three times higher than that of 70-kDa Dextran (0.73 ± 0.1 μg/mL for 10 kDa vs. 0.25 ± 0.01 μg/mL for 70 kDa, *p*=0.05). After 2 hours, this difference increased to a five-fold higher concentration for the smaller molecule (1.7 ± 0.28 μg/mL for 10 kDa vs. 0.3 ± 0.01 μg/mL for 70 kDa, *p* < 0.0001), confirming size-selective permeability and demonstrating effective compound exchange across the CAM–device interface based on molecular size (Figure 5(c)).

**Figure 6:**
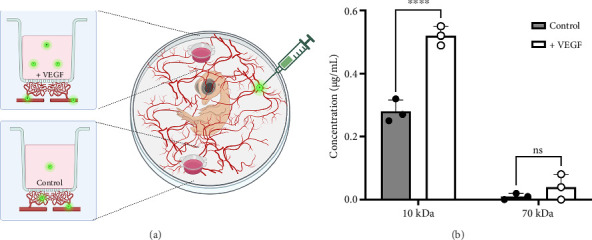
Effect of VEGF on dextran diffusion in the CAM. (a) Two inserts were placed in the same embryo, one containing 0.5 μg/mL VEGF and the other containing only PBS. The 10-kDa or 70-kDa dextran was then injected into the circulation. (b) Concentration of 10-kDa and 70-kDa dextran on the inserts 30 min after injection. Inserts containing VEGF showed a 3-fold higher dextran diffusion compared to the control inserts without VEGF. Two-way ANOVA, mean ± SD. ns = *p* > 0.05, ^∗∗∗∗^*p* < 0.0001. *n* = 3 samples per condition and time point.

Well-controlled permeability of the vasculature in our membranous device is relevant for tissue function. To further modulate vascular permeability, 0.5 μg/mL of VEGF was apically applied to one of the two inserts placed on the same CAM (Figure 6(a)). After 3 days, inserts treated with VEGF exhibited increased vascular permeability and enhanced diffusion of small molecules (Figure 6(b)). Embryos injected with 10-kDa dextran displayed significantly higher concentrations in the VEGF-treated inserts (0.52 ± 0.03 μg/mL) compared to the PBS control inserts (0.28 ± 0.03 μg/mL) (*p* < 0.0001). Although embryos injected with 70-kDa dextran showed a trend toward greater diffusion in the VEGF-treated inserts, this difference was not statistically significant. These results suggest that VEGF enhances permeability for small molecules without compromising membrane selectivity.

### 3.3. Functional Epithelial Cells Can Be Maintained in Membranous Devices on the CAM

**Figure 7:**
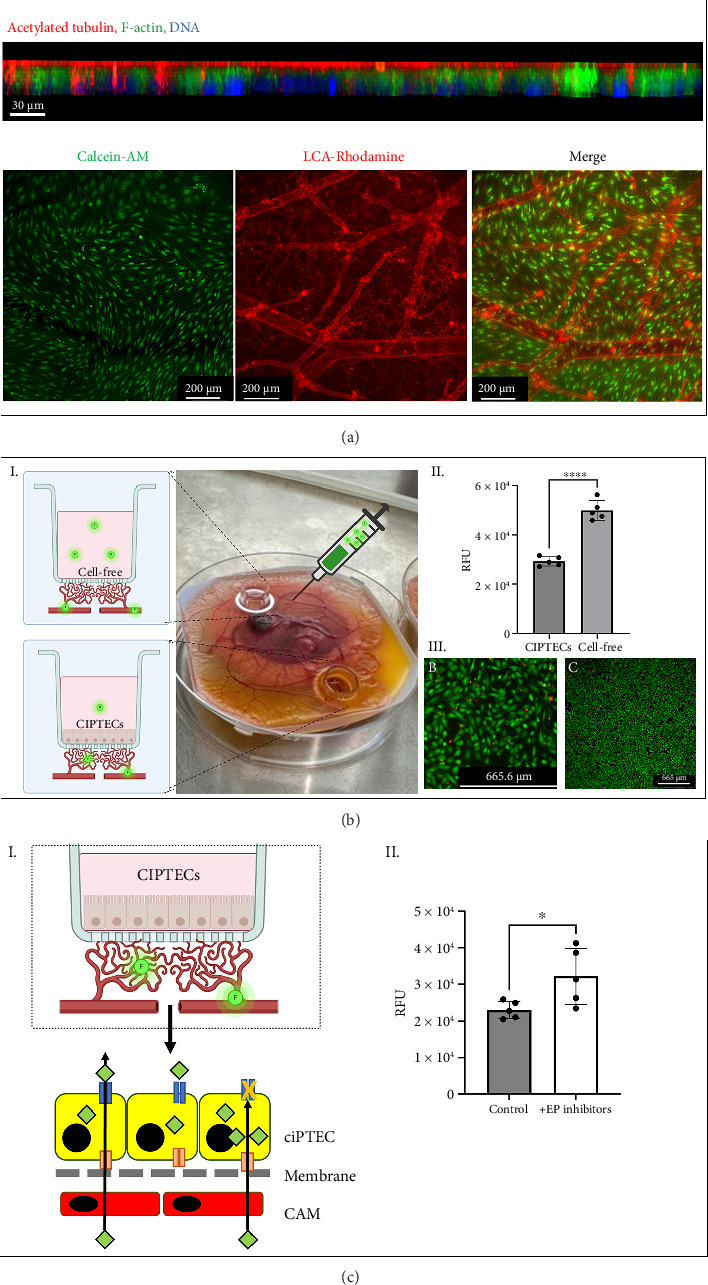
Characterization of conditionally immortalized proximal tubular epithelial cells (ciPTEC) cultured on the CAM model. (a) Representative cross-section of the ciPTEC monolayer on the insert placed on the CAM, showing polarized cells. The image shows acetylated tubulin (cilia, red), F-actin (green), and DNA (blue). The bottom row shows Calcein-AM staining of live cells on the inserts after 4 days of culture, and Lens Culinaris Agglutinin (LCA-Rhodamine) staining of the underlying chick endothelium and vasculature. (b) Barrier integrity assessment of the ciPTEC monolayer on the CAM. (I) Image showing two inserts, one with ciPTEC and one cell-free control, after injection of fluorescent inulin tracer into the circulation. (II) Quantification of the fluorescent signal in the inserts after 30 min, showing significantly lower permeability in the ciPTEC-containing insert (*n* = 5, One-way ANOVA, ^∗∗∗∗^*p* < 0.0001). (III) Live-dead staining of the ciPTEC monolayer after 4 days on the CAM. (c) Functional evaluation of efflux transporter activity in ciPTEC on the CAM. (I) Schematic of the fluorescein-based organic anion transporter (OAT) assay, with yellow representing ciPTEC, red the CAM endothelium, green the fluorescein substrate, orange the OAT1-3 transporters, and blue the efflux pumps BCRP and MRP. (II) Intracellular accumulation of fluorescein in control inserts and inserts treated with efflux pump inhibitors (*n* = 5, one-way ANOVA, ^∗^*p* < 0.05).

ciPTEC were cultured, seeded onto the upper side of the double-coated membrane of the inserts, and expanded until confluency and differentiation. The inserts were then positioned directly above a highly vascularized area of the CAM for 4 days, maintaining a proximity of less than 50 microns from the capillary bed. Following implantation, the embryo survival rate was high, exceeding 70%. Live-dead assays confirmed that ciPTEC remained viable after the incubation on the CAM for at least 4 days. Cross-section imaging demonstrated that the monolayer was polarized, with apical cilia stained with acetylated tubulin, indicating healthy and well-organized cell morphology (Figure 7(a)).

### 3.4. Tubular Epithelium Maintains Selective Barrier and Transport Functions in Vascularized Membranes

For the functional application of kidney epithelialized membranes, both a proper barrier function and selective transport function are required. Therefore, we evaluated whether cellularization of the membrane reduced the passive diffusion of known exogenous compounds injected into the circulation. Cellularized inserts reduced the diffusion of inulin-FITC by 40% compared to cell-free inserts (*p* < 0.0001), indicating functional tight junction formation and reduced passive permeability (Figure 7(b)). It is of note that inulin is a compound that is not handled by the tubular epithelial cells in vivo [40, 41] but excreted by filtration solely.

One of the key functions of proximal tubular cells is the excretion of endogenous metabolites (uremic toxins) via the OATs at the basolateral side and multidrug resistance protein 4 (MRP4) and breast cancer resistance protein (BCRP) at the luminal side [42, 43]. To evaluate the functionality of these transporters in our model, we used the organic anionic substrate fluorescein. Following intravascular injection, fluorescein was taken up by the renal epithelial cells seeded in the inserts, resulting in an average intracellular fluorescence of 23 ± 2.3 × 10^3^ RFU. After uptake, we would expect a transepithelial clearance of the fluorescein, mediated via the efflux pumps MRP4 and BCRP [44]. To verify this mechanism, we inhibited MRP4 and BCRP with MK571 and KO-143, respectively, which led to a 28% increase in intracellular fluorescein accumulation (*p* < 0.05) (Figure 7(c)). These data demonstrate that ciPTEC on vascularized membranes retain transporter expression and function, enabling polarized vectorial transport.

## 4. Discussion

To our knowledge, we are the first to present the CAM model as a tool to develop and evaluate crucial aspects of CMD. We demonstrated that the membranes placed on the CAM become progressively vascularized, and that both structural and functional vascularization can be modulated by adapting the matrix interface and by growth factor supplementation. This level of control is essential in CMD development, where vascular proximity and perfusion directly influence nutrient delivery, waste removal, and long-term graft viability. Importantly, we found that membrane vascularization could be enhanced using a collagen-fibrin hydrogel, particularly when supplemented with VEGF. Quantitative image analysis revealed significant increases in TVL, NVJ, and vasculogenic index—key metrics that indicate robust angiogenesis. This is consistent with previous reports that matrix and growth factor environments can drive neovascular patterning in the CAM, and supports its suitability for testing biomaterial–vascular interactions in a preclinical context [45–49]. Moreover, SRUS imaging confirmed that the CAM vasculature was not only morphologically mature but functionally perfused, as demonstrated by directional MB flow and consistent dye distribution.

A significant finding of this work is the ability of the CAM model to evaluate the molecular barrier properties of CMD membranes. Membranes excluded larger molecules like 70-kDa dextran and prevented host cell infiltration, while allowing free diffusion of smaller compounds such as 10-kDa dextran. These data confirm that the semipermeable membrane retains selective permeability and functions as an immune-isolating barrier, a central requirement for CMD applications [50, 51]. The inability of cells to penetrate the insert unless the membrane was mechanically compromised further reinforces the robustness of the barrier function.

Our work expands the application of the CAM model beyond its traditional use in angiogenesis, drug research, tissue engineering, biocompatibility testing, xenograft applications and organoid research [20, 52–56]. While many of these applications rely on quantitative assessments (angiogenesis quantification, cell migration and invasion (or lack thereof), tissue responses (such as foreign body reaction), blood flow dynamics, pharmacodynamics, and pharmacokinetics), we introduce novel readouts tailored to CMD development, such as barrier function and selective compound transport across the epithelialized membrane, by adapting existing in vitro models for in vivo use in the CAM assay [57, 58]. This adds depth to CMD testing by enabling assessment of not just structure but also performance.

Historically, CMD have been explored for organ function replacement for nearly half a century [16]. Early efforts in the 1980s focused on CMD for various organ systems, including the pancreas, liver, and brain [59–61]. More recently, CMD have been developed predominantly for diabetes management [62–64], with animal models, particularly rodents, serving as the primary evaluation platforms [15]. The CAM model presented here introduces two significant innovations to this field. First, it enables real-time control and quantification of CMD vascularization in a living system. Second, it incorporates an epithelialized membrane, facilitating specific, polarized transport by cells seeded in the CMD. Together, these capabilities provide a dynamic experimental platform for rapidly iterating the functional properties of CMD designs prior to more complex animal studies.

In the context of renal replacement therapies (RRT), our study support CMD as promising complement to extracorporeal and implantable technologies. Multiple biophysical approaches aim to enhance hemodialysis, such as hemodiafiltration and sorbent-based dialysis membranes, which improve the removal of larger and protein-bound toxins [65, 66]. Our study complements these innovations by providing a versatile in vivo platform for testing transmembrane transport and vascularization in implantable filtration-based kidney devices. CMD also align conceptually with bioartificial kidney (BAK) technologies, such as those developed by Humes et al., which also employ renal epithelial cells to restore specific metabolic functions [67]. However, while the BAK relies on a perfusion system with larger pore membranes for diffusion-based clearance, CMD are designed for integration with the host microvasculature and feature epithelialized membranes enabling polarized, active transport.

Vascularization is not strictly necessary for extracorporeal organ replacement therapy like dialysis and extracorporeal membrane oxygenation, as these systems can connect directly to large blood vessels, akin to current hemodialysis methods [68, 69]. In contrast, intracorporeal CMD offer a potentially less invasive and more continuous approach. Traditional intracorporeal designs involve surgical connections to large arteries, but CMD could alternatively be implanted in highly vascularized areas, such as subcutaneous tissue. This approach mirrors the principle of peritoneal dialysis, wherein the peritoneal cavity serves as a natural CMD, enabling adjustable fluid exchange for optimized clearance [70]. A cell-based implantable CMD would merge continuous clearance with metabolic functions, advancing the biomimetic potential of kidney replacement therapies.

The choice of cells in the CMD is an important consideration. In this study, ciPTEC were used due to their established safety and functionality in BAK research. ciPTEC form stable monolayers on porous membranes and demonstrate effective transport and endocrine activities, including vitamin D activation [58, 71]. Furthermore, they exhibit low immunogenicity and tumorigenicity, making them a viable option for RRT [72]. Beyond cell lines like ciPTEC, organoids hold promise for CMD [73–75]. These can be derived from iPSCs as well as from adult stem cells, and offer an interesting source of functional epithelial cells. Organoid-derived epithelial cells offer extensive proliferative capacity, long-term culture stability, and near-mature expression of essential enzymes and transporters [75, 76]. Although proper structural and functional organization of the organoid cells remains a challenge, CMD membranes can provide biochemical and topological cues to guide their differentiation and functional integration, enhancing therapeutic potential.

One of the major advantages of the CAM model is its markedly lower cost and shorter experimental duration compared to rodent models. The fertilized chicken eggs used in these experiments cost approximately €0.50 per egg. No specialized animal housing, breeding, or extensive regulatory approval is required, which further reduces expenses and logistical barriers [77, 78]. By contrast, a single immunodeficient mouse typically costs ∼€53.8, and when including food and housing for 2 weeks, the cost per mouse rises to ∼€69.5 [79]. Longer experimental timelines, additional facility maintenance, and regulatory compliance further increase the total cost of rodent studies [80]. In terms of duration, CAM experiments can be completed within 10–14 days, enabling rapid assessment of growth and therapeutic interventions, while rodent models typically require several weeks to months.

Our findings affirm the CAM model's advantages for CMD development while acknowledging its limitations. As an embryonic avian model, it differs from adult mammalian systems in several aspects, including in immune system development, vascular structure, and metabolic pathways [81–84]. Although this species difference must be taken into account, the relevance of the model to the human situation does appear from its frequent application in the study of human tissue engraftment, particularly in cancer research and tissue engineering [85–89]. Another drawback is the relatively short experimental window, which lasted at maximum 7 days in our experiments. Although it could be argued that this still covers a substantial part of the host animal's development stages, chronic host reactions or cell function beyond this time window cannot be assessed in the CAM alone. Further validation in extended-duration models would be necessary to confirm sustainability of the observed effects. The CAM is expected to complement other animal models for longer-term studies, bridging the gap between in vitro and in vivo research. A final limitation relates to the fluorescein transport assay: while fluorescein is widely used as a functional substrate of renal OATs, it does not allow isoform-specific attribution of transport [37, 90]. Future studies using additional substrates such as para-aminohippurate or estrone sulfate, combined with transporter expression profiling, will be important to strengthen the physiological interpretation of vectorial transport in this model.

As the population ages, the burden of organ failure is reaching endemic proportions. The rapid development in biomaterials and cell-based technologies has positioned CMD as promising avenue for functional organ replacement. In this study, we demonstrate that the CAM model offers a powerful and practical platform to optimize and evaluate key parameters of CMD, including epithelial membrane–specific barrier and selective transport functions. The CAM effectively bridges the gap between in vitro assays and traditional animal models, enabling high-throughput, cost-efficient preclinical testing. As such, it holds significant potential to accelerate the development and refinement of CMD toward clinical application in the treatment of organ failure.

## Data Availability

The data that support the findings of this study are available from the corresponding author upon reasonable request.

## Supporting Information

Supporting Information 1 Figure S1: Timeline of the ex ovo CAM model. The key events are: Day 0: Egg incubation—The embryo development starts inside the intact eggshell. Day 3: Embryo transfer—The embryo is carefully removed from the eggshell and placed in a specialized culture dish. Day 10: Insert incubation—a semipermeable membrane insert is placed on top of the CAM, allowing the integration and interaction of cells/tissues with the vascular network. Day 14: Experiments—After the insert has become vascularized, various experiments can be performed, such as evaluating compound diffusion, or cell function.

Supporting Information 2 Figure S2: Increased cell presence on inserts with punctured semipermeable membranes. The bar graph compares the number of cells on control inserts versus inserts with punctured membranes. Puncturing the membrane led to a substantial increase in cell presence on the inserts. A: Contents of the insert with intact membrane. B: Contents of the insert with pre-drilled holes (*n* = 3, mean ± SD, *t*-test, ^∗∗^*p* < 0.01).

Supporting Information 3 Supporting Video 1: Bright-field live imaging at high-magnification (40x) recording of the chorioallantoic membrane (CAM), highlighting a central vessel (red) with smaller converging vessels on either side and erythrocytes flowing through the CAM.

Supporting Figure 1 provides a comprehensive overview of the experimental timeline and the key procedural milestones in the chick chorioallantoic membrane (CAM) assay. The figure illustrates the progression from initial egg incubation (ED0) through embryo transfer to the ex ovo system (ED3), placement of the cell culture insert onto the CAM (ED10), and the final experimental endpoints (ED14). Accompanying images document the morphological development of the embryo and CAM vasculature at these critical time points, thereby contextualizing the experimental framework and the stages at which interventions and analyses were performed.

Supporting Figure 2 presents quantitative data comparing cellular presence on semipermeable membrane inserts with intact membranes versus those with predrilled perforations. This figure supports the conclusion that physical modification of the membrane significantly enhances cell infiltration, an important consideration for optimizing membrane design and permeability in the context of cell macroencapsulation devices.

Supporting Video 1 offers a high-magnification bright-field time-lapse visualization of the CAM vascular network. The video captures the dynamic flow of erythrocytes within central and branching blood vessels, illustrating the active and functional state of the vascular bed. This dynamic imaging substantiates the CAM model's suitability for investigating vascular integration and perfusion in bioengineered constructs.
