# Supplementary figures and images for: The Chorioallantoic Membrane as a Platform for Developing Vascularized Cell Macroencapsulation Devices
Source: J Tissue Eng Regen Med. 2025 Nov 15;2025:5577199. doi: 10.1155/term/5577199 (PMC12640263; doi:10.1155/term/5577199)

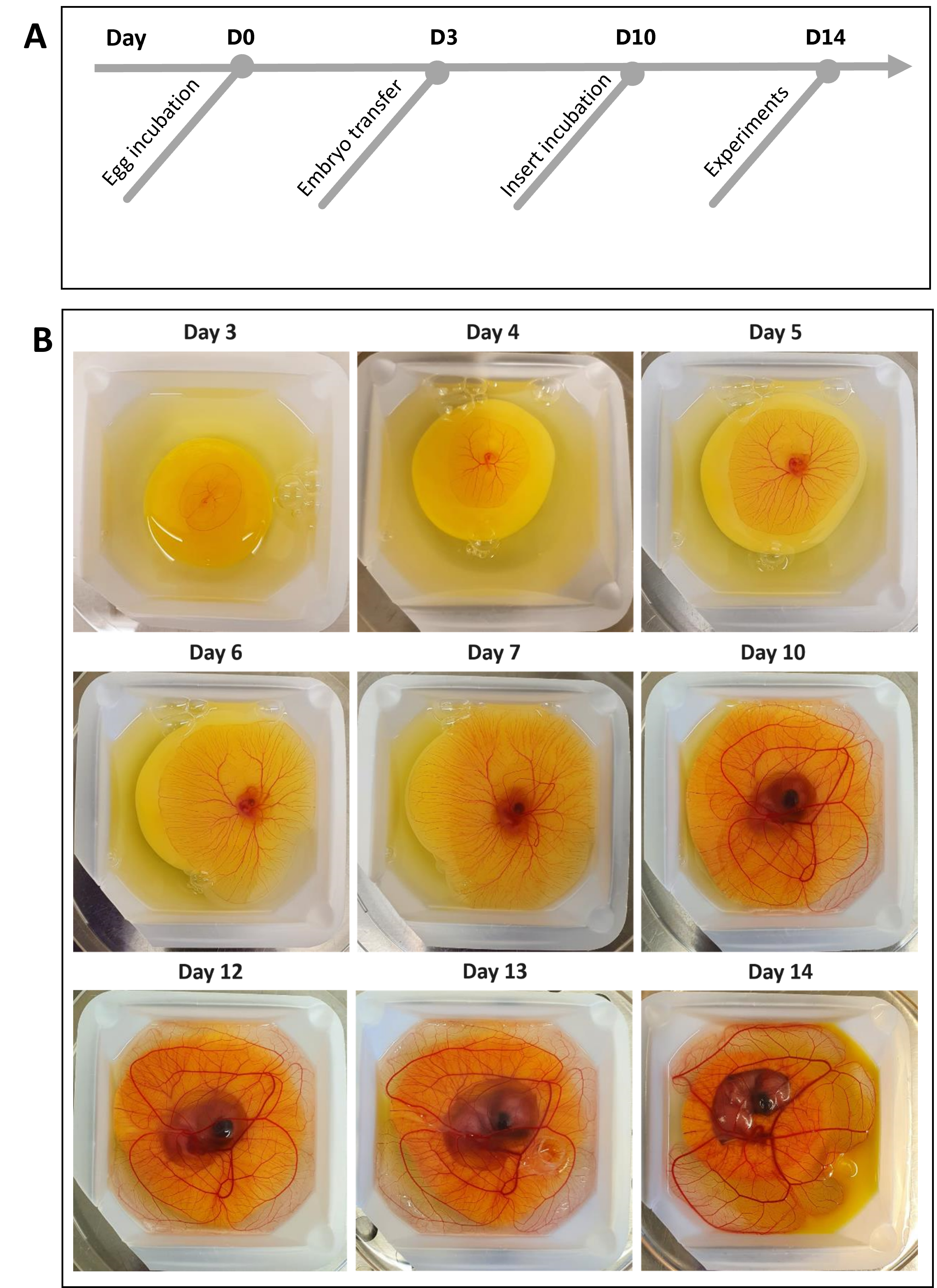

Supplement: Supporting Information 1 — Figure S1: Timeline of the ex ovo CAM model. The key events are: Day 0: Egg incubation—The embryo development starts inside the intact eggshell. Day 3: Embryo transfer—The embryo is carefully removed from the eggshell and placed in a specialized culture dish. Day 10: Insert incubation—a semipermeable membrane insert is placed on top of the CAM, allowing the integration and interaction of cells/tissues with the vascular network. Day 14: Experiments—After the insert has become vascularized, various experiments can be performed, such as evaluating compound diffusion, or cell function. [file 5577199.f1.png]

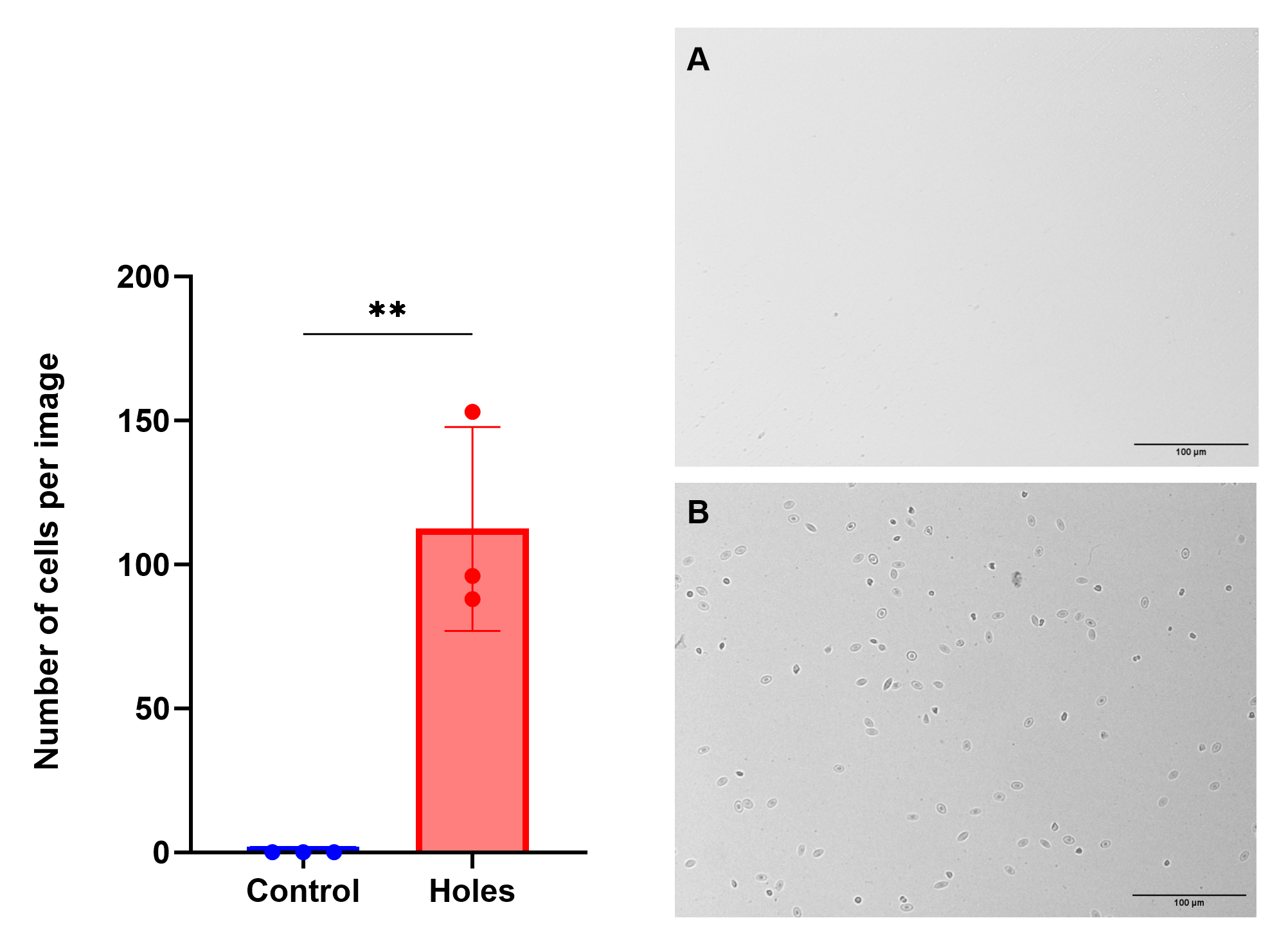

Supplement: Supporting Information 2 — Figure S2: Increased cell presence on inserts with punctured semipermeable membranes. The bar graph compares the number of cells on control inserts versus inserts with punctured membranes. Puncturing the membrane led to a substantial increase in cell presence on the inserts. A: Contents of the insert with intact membrane. B: Contents of the insert with pre-drilled holes (n = 3, mean ± SD, t-test, ∗∗p < 0.01). [file 5577199.f2.tif]
